# Value of Perfusion CT in the Prediction of Intracerebral Hemorrhage after Endovascular Treatment

**DOI:** 10.1155/2021/9933015

**Published:** 2021-07-22

**Authors:** Friederike Austein, Antonia Carlotta Fischer, Jens Fiehler, Olav Jansen, Thomas Lindner, Susanne Gellißen

**Affiliations:** ^1^Department for Diagnostic and Interventional Neuroradiology, University Medical Center Hamburg-Eppendorf, Hamburg, Germany; ^2^Department of Radiology and Neuroradiology, University Hospital Schleswig-Holstein, Germany

## Abstract

**Background:**

Intracerebral hemorrhage (ICH) is a serious complication of endovascular treatment (EVT) in stroke patients with large vessel occlusion (LVO) and associated with increased morbidity and mortality.

**Aims:**

Identification of radiological predictors is highly relevant. We investigated the predictive power of computed tomography perfusion (CTP) parameters concerning ICH in patients receiving EVT.

**Methods:**

392 patients with anterior circulation LVO with multimodal CT imaging who underwent EVT were analyzed. CTP parameters were visually evaluated for modified ASPECTS regions and compared between patients without ICH, those with hemorrhagic infarction (HI), and those with parenchymal hematoma (PH) according to the ECASS criteria at follow-up imaging and broken down by ASPECTS regions.

**Results:**

168 received intravenous thrombolysis (IV-rtPA), and 115 developed subsequent ICH (29.3%), of which 74 were classified as HI and 41 as PH. Patients with HI and PH had lower ASPECTS than patients without ICH and worse functional outcome after 90 days (*p* < 0.05). In 102 of the 115 patients with ICH, the deep middle cerebral artery (MCA) territory was affected with differences between patients without ICH, those with HI, and those with PH regarding cerebral blood volume (CBV) and blood-brain barrier permeability measured as flow extraction product (FED) relative to the contralateral hemisphere (*p* < 0.05). Patients with PH showed larger perfusion CT infarct core than patients without ICH (*p* < 0.01).

**Conclusion:**

None of the examined CTP parameters was found to be a strong predictor of subsequent ICH. ASPECTS and initial CTP core volume were more reliable and may be useful and even so more practicable to assess the risk of subsequent ICH after EVT.

## 1. Introduction

The HERMES [1] individual patient meta-analysis of trials published in 2015 showed clinical benefits for endovascular treatment (EVT) in a multitude of patients, suggesting that benefit from EVT is predictive to a broad range of acute ischemic stroke (AIS) patients with large vessel occlusion (LVO). Subsequent intracerebral hemorrhage (ICH) due to hemorrhagic conversion is a major complication after EVT, especially in intracranial hematoma [2]. Various predictors of ICH after intravenous thrombolysis (IV-rtPA) have been reported [3]. The European Cooperative Acute Stroke Study (ECASS) II subdivides ICH into hemorrhagic infarction (HI) and parenchymal hematoma (PH) [4]. The incidence of ICH varies from 9% to 25% [5–9]. At the same time, mortality at 90 days did not significantly differ, and the risk of PH and symptomatic ICH was not higher with EVT than that with other therapies [1]. According to Nogueira et al. [2], both HI and PH were independent predictors of poor long-term functional outcome. Others described that only PH was associated with increased risk for death or disability [10]. Since EVT is an established therapy for patients with LVO, optimal patient selection for safe and efficient treatment is an important goal [11]. The knowledge of variables that allow the prediction of ICH may contribute to prognosis. The Alberta Stroke Program Early CT Score (ASPECTS) is considered a predictor of functional outcome and ICH [12]. Many authors assessed the value of CT perfusion (CTP) regarding the prediction of ICH in AIS and tried to determine CTP parameters in the prediction of ICH, e.g., cerebral blood flow (CBF), cerebral blood volume (CBV), and blood-brain barrier permeability measured as flow extraction product (FED) [13–16].

Most results are limited by small statistical power due to small study populations (41-146 patients) [13, 15].

## 2. Hypothesis

In this large well-selected study cohort, we want to confirm with previous published hypotheses that common clinically used CT parameters are reliable predictors of subsequent ICH after EVT.

## 3. Methods

A retrospective analysis of prospectively collected data was performed for all patients presenting to our tertiary care academic comprehensive stroke center with AIS and LVO in the anterior circulation treated with EVT between January 2010 and March 2017. The local ethics committee approved the study and waived informed consent. The study was conducted in accordance with the regulations of the 1964 Helsinki declaration and later amendments.

### 3.1. Patient Cohort

**Figure 1:**
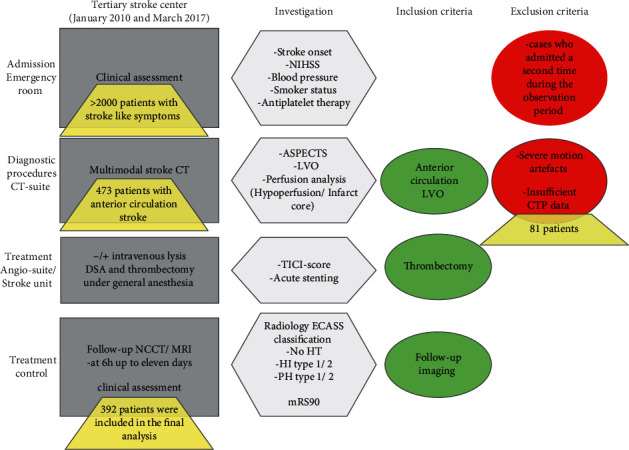
The flowchart shows the stroke workflow in our tertiary stroke center with standardized clinical and imaging assessment, treatment, and follow-up and also the finally included patients.

Patient inclusion criteria were (1) AIS, (2) performance of initial non-contrast-enhanced CT (NCCT), (3) CT angiography (CTA) to determine the localization of the vessel occlusion, (4) CTP, and (5) posttreatment follow-up imaging within 6 hours up to eleven days. No exclusion based on age or clinical severity was done. According to standard guidelines, eligible patients additionally received IV-rtPA. The standardized stroke workflow with clinical and imaging assessment, treatment, and follow-up is summarized in Figure 1.

Patient characteristics and clinical and treatment-associated variables were extracted from the medical records, including age, sex, NIHSS on admission, time from symptom onset to admission, ASPECTS, reperfusion grade (thrombolysis in cerebral infarction (TICI)), and the modified Rankin Scale after 90 days (mRS90d).

### 3.2. Preprocedural Imaging Evaluation

**Figure 2:**
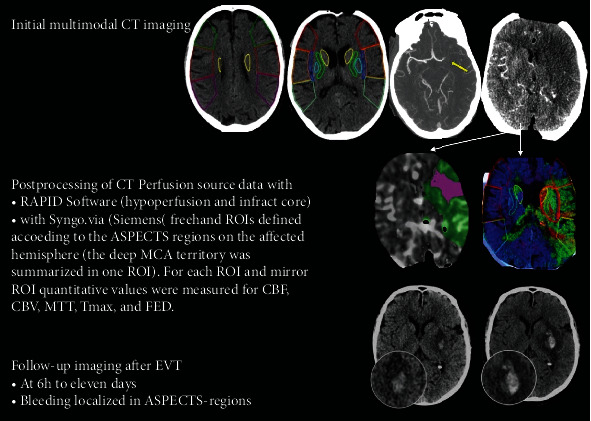
Visualization of the initial imaging, postprocessing of perfusion data, and follow-up. Abbreviations: ASPECTS, Alberta Stroke Program Early CT Score; CBF, cerebral blood flow; CBV, cerebral blood volume; EVT, endovascular treatment; FED, flow extraction product; MCA, middle cerebral artery; MTT, mean transit time; ROI, region of interest; and Tmax, maximum enhancement time.

Each patient was examined with a NCCT to exclude ICH and to estimate the volume of irreversible infarction tissues. Vessel occlusions were confirmed by CTA. Imaging workflow and CTP post-processing and follow-up are visualized in Figure 2.

### 3.3. CTP Image Acquisition

All CT scans were performed using a 64-slice CT scanner equipped with a 40 mm wide detector (Brilliance 64, Philips Medical Systems, the Netherlands; imaging parameters: 80 kVp, 150 mAs, and 32 × 1.25 mm detector collimation, duration: 60 seconds). After NCCT, CTP was conducted with the toggling table technique, allowing coverage of the brain of 80 mm. A scan delay of 3 seconds was applied after injecting 60 mL (5 mL/s) of iodinated contrast agent (350 mg I/mL Iomeron® 350, Bracco Imaging, Germany).

### 3.4. CTP Image Postprocessing and Analysis

Two different packages of processing software were used. Details of the software settings can be found in the Supplementary Material (available here).

#### 3.4.1. Package A

CTP images were processed with syngo (CT Neuro Perfusion (Siemens Healthcare, Erlangen, Germany)). This semiautomated software package requires the process of registration and segmentation. We chose the deconvolution model with a delay-insensitive algorithm [17, 18]. The Patlak model [19], which was based on a two-compartment model, was used for assessing the flow extraction product (FED). More details can be found in Supplement S1.

#### 3.4.2. Package B

RAPID (iSchemaView Inc., Menlo Park, California, USA) is an automated software allowing online estimation of perfusion maps and mismatch using a delay-insensitive algorithm. More details can be found in Supplement S2.

For consistency of data evaluation, only one investigator (AC. F.) performed all CTP imaging studies which were controlled by a neuroradiologist (F. A.) with at least 7 years of experience in stroke imaging.

### 3.5. Stroke Treatment Protocol

Clinical routine indications for EVT in our center were AIS with substantial neurological deficits, National Institutes of Health Stroke Scale (NIHSS) > 6, a target LVO, lack of early ischemic infarct signs (ASPECT Score ≥ 5), and/or perfusion imaging indicating a mismatch. Thrombolysis in cerebral infarction (TICI) reperfusion scores of 2b or 3 were defined as successful.

### 3.6. Follow-Up

The primary outcome of this study was any new ICH diagnosed at follow-up imaging. On the follow-up scan, ICH was classified according to the radiological ECASS criteria only [20]. ICH was defined as HI and PH which were further classified as HI 1 as small petechiae along the peripheral margins of the infarcted area, HI 2 as confluent petechiae within the infarct but without space-occupying effect, PH 1 as hematoma with a mass effect of ≤30%, and PH 2 as hematoma with a mass effect of >30% of the infarcted area [4].

### 3.7. Statistical Analysis

Initially, all data were descriptively broken down by groups of patients without ICH and with HI 1, HI 2, PH 1, and PH 2.

As the distinction between HI and PH according to the ECASS II grading system appears to be clinically relevant [21], it was applied in our study to divide the patients into groups for further analysis. Patients with HI 1 and HI 2 were generalized as patients with HI, and patients with PH 1 and PH 2 as patients with PH.

Prior to the analysis, it was determined whether the data follows a normal distribution to choose the appropriate parametric or nonparametric tests.

Categorical variables are presented proportionally, and due to the data distribution, quantitative variable results are presented as medians with corresponding interquartile ranges (IQR). IV-rtPA treatment status, TICI score, and application of stents were compared between the three groups using the Pearson chi-squared test.

Since age, NIHSS on admission, ASPECTS, time from symptom onset to admission, and mRS90d were not normally distributed, the Kruskal-Wallis *H* test was used. For statistically significant differences (*p* < 0.001), post hoc analysis with the Mann-Whitney *U* test was performed for comparing only two groups with *p* values < 0.05 being considered statistically significant.

Volumes of ischemic core and hypoperfused tissue were also compared by using the Kruskal-Wallis *H* test, and for statistically significant differences (*p* < 0.05), post hoc analysis was performed.

For all the patients collectively and subdivided into patients without ICH, with HI 1, HI 2, PH 1, and PH 2, the ROIs were compared regarding their frequencies of being affected by hemorrhage using the Cochran *Q* test. For statistically significant differences (*p* < 0.001), post hoc analysis was performed between each two regions of interest with McNemar's test with *p* values < 0.05 being considered statistically significant.

For the ROI in the deep MCA territory, CBF, CBV, Tmax, and FED were compared using the Mann-Whitney *U* test with a 5% significance level.

The effect sizes of parameters that showed statistically significant differences between the groups were determined using Cohen's *d* with a pooled standard deviation.

A multivariate logistic regression analysis was not applicable due to the data distribution.

Statistical analyses were performed with IBM SPSS Statistics 23 (SPSS Inc., an IBM Company, Chicago, IL).

## 4. Results

### 4.1. Patient Characteristics and Outcome

392 of 473 patients were included in the final analysis. 81 patients were excluded because of insufficient or nonusable data.

Mean age was 73 (64-80) years (204 female, 188 male), and median baseline NIHSS score was 14. 183 patients (46.9%) had an occlusion of the M1 segment of the MCA, 49 (12.6%) of the ICA bifurcation, 38 (9.7%) of the M2 segment of the MCA, and 18 (4.6%) of the ICA. 59 patients (15.1%) had two sites of occlusion, and 31 (7.9%) three or more. 12 (3.1%) had various occlusions in the anterior circulation.

Of the 392 enrolled patients with AIS, 168 (42.9%) received IV-rtPA. Of the 392 patients, 115 patients (29.3%) had ICH detected in the follow-up imaging: 19 (4.8%) with HI 1, 55 (14%) with HI 2, 18 (4.6%) with PH 1, and 23 (5.9%) with PH 2. No significant differences were found between the groups (without ICH, HI, and PH) regarding age, gender, blood pressure, blood glucose, hypercholesterolemia, antithrombotic medications before stroke, treatment with IV-rtPA, or acute carotid stenting.

The median time between admission and initial manifestation of ICH and the median time between admission and the largest manifestation of ICH in the follow-up imaging were one day. In 75% of the patients with HI 1 and HI 2, initial manifestation of ICH occurred within two days after admission, whereas in 75% of patients with PH 1 or PH 2, initial manifestation of ICH occurred within the first day. In 25% of patients with HI 2 and PH 1, the largest manifestation of ICH occurred three days and more after admission (Supplement Figure I).

In summary, there were 74 patients (18.9%) with HI and 41 patients (10.5%) with PH. Patients with HI (*p* = 0.001) and PH (*p* = 0.001) had a significantly worse functional outcome in comparison to patients without ICH. Patients with PH had a significantly worse outcome than patients with HI (*p* < 0.05). Patient characteristics are summarized in Table 1.

### 4.2. Imaging Findings and CT Parameters

The median ASPECTS was 8 (IQR 7-10), and the median infarct core volume was 5 mL (IQR 0-18). The ASPECTS was significantly lower in patients with HI (*p* = 0.001) and PH (*p* = 0.005) than in patients without ICH. Also, patients with PH showed a significantly larger infarct core than patients without ICH (*p* = 0.009) (Table 2).

#### 4.2.1. Subsequent Intracerebral Hemorrhage regarding the ASPECTS ROIs

There was a significant difference in the rate of ICH between different anatomical ROIs.

In 102 of the 115 patients with ICH, the deep MCA territory was affected. The cortical regions were affected markedly less often. Therefore, we investigated whether there was a difference regarding the CTP parameters in the deep MCA territory between patients without ICH, those with HI, and those with PH.

#### 4.2.2. CTP Parameters of the ASPECTS ROIs

In all groups (ECASS II) and in all ROIs (ASPECTS), CBF was lower, Tmax was considerably higher, and FED was higher on the affected than the contralateral hemisphere. CBV was sometimes lower and sometimes higher on the affected hemisphere.

The absolute values of CBV in the deep MCA territory on the affected hemisphere were significantly lower than in the cortical ASPECTS ROIs.

#### 4.2.3. CTP Parameters in the Deep Middle Cerebral Artery Territory

**Figure 3:**
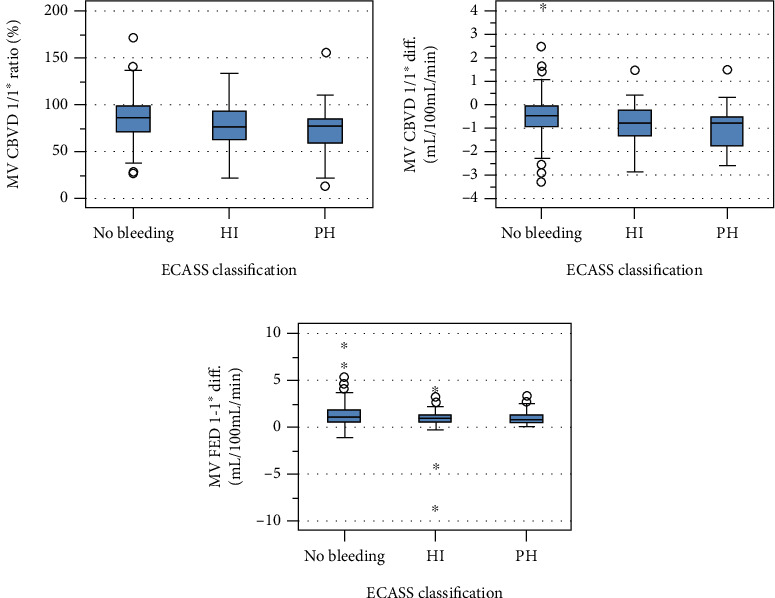
(a) Mean values of the CBV in the deep MCA ROI of the affected hemisphere relative to the contralateral hemisphere in percent (1/1^∗^ ratio). (b) Absolute differences between the mean values of the CBV in the deep MCA ROI of the affected hemisphere and the contralateral hemisphere (1-1^∗^ Diff.). (c) Absolute differences between the mean values of the FED in the deep MCA ROI of the affected hemisphere and the contralateral hemisphere (1-1^∗^ Diff.). Abbreviations: CBV, cerebral blood volume, FED, flow extraction product; HI, hemorrhagic infarction; PH, parenchymal hematoma; MCA, middle cerebral artery; and ROI, region of interest.

In the deep MCA territory, patients with HI and PH showed significantly lower CBV on the affected hemisphere, significantly lower ratios of CBV of the affected relative to the contralateral hemisphere, significantly higher absolute differences of CBV, and significantly lower absolute differences of FED between the affected and the contralateral hemisphere than patients without ICH. Additionally, patients with HI had a significantly lower ratio of FED of the affected hemisphere than patients without ICH. Patients with PH had significantly lower ratios and significantly higher absolute differences of CBF of the affected hemisphere than patients without hemorrhage. There were no statistically significant differences between patients with HI and PH (Supplement Table 1). However, there was a large statistical scattering of the results (Figure 3). Presumably, there was no effect of the absolute differences of CBV and FED for the deep MCA territory ROIs and their contralaterals regarding the distinction between patients without ICH, those with HI, and those with PH and only a small effect of the ratio between the CBV on the affected hemisphere for distinction between patients without ICH and those who develop HI and PH.

## 5. Discussion

To our knowledge, taking into consideration the 392 well-selected AIS patients who underwent EVT with 115 detected cases of ICH in the follow-up imaging, our study is the largest single-center study for this purpose. Our study confirms that the incidence of ICH was similar in patients receiving bridging therapy or direct thrombectomy [22]. Nevertheless, the underlying pathophysiological mechanisms may differ, as there is evidence of a neurovascular toxic effect of IV-rtPA, which leads to the disruption of the blood-brain barrier. This effect seems to be less pronounced with a mechanical thrombectomy [23].

Evaluating the predictive power of single CTP parameters regarding the development of ICH, we found the absolute and relative differences of CBV and the absolute differences of FED of the affected hemisphere to be statistical significantly different between patients without ICH, compared to those with HI and PH. However, as it becomes evident looking at the boxplots (Figure 3), there was a large statistical dispersion and a presumably small effect of that finding. The number of cases in our study with bleeding events is higher than in all other published studies—to the best of our knowledge. Still the statistical power is low because of the overall small number of ICH events and should be looked at critically to avoid the “winner's curse,” which reflects the acceptance of a small statistically significant *p* value which results by low-powered studies. Being lucky enough to discover a true effect, researchers are more likely to exaggerate the size of that effect. When small low-powered studies do claim a discovery, that discovery is more likely to be false. Therefore, we cannot confirm the results of previous studies that evaluated single CTP parameters as promising for the prediction of ICH. As previous authors mentioned, the rarity of the occurrence of ICH and especially PH, the small sample sizes of patients who fit the inclusion criteria, and the exploration of various predictor variables involve the risk of statistical models overfitting and consequent type 1 errors [24]. Larger sample sizes and more robust statistical techniques are required to determine the risk of ICH [24]. The mentioned authors themselves described several limitations concerning their studies, including small sample sizes, low positive predictive value, and high negative predictive value, suggesting severe ischemia to be a necessary but not a sufficient mediator for PH [15] and BBB damage accounting only for 10% of the ICH grade [21]. A meta-analysis regarding CTP for prediction of ICH in AIS, including a total of 1134 patients, described the presence of heterogeneity and differences in cut-off values for CTP parameters across the included studies. Furthermore, the authors found a high likelihood of publication bias, presumably due to the circumstance that studies with a statistically significant result are more likely to be published and elucidate the possibility that the diagnostic performance of perfusion CT for the prediction of ICH therefore might have been overestimated [25].

In our study, ICH occurred most commonly in the deep MCA territory. CBV in the deep MCA territory of the affected hemisphere showed lower median values compared to cortical regions. Low relative CBV appears to be associated with worse collateral circulation and represents an independent predictor of infarct growth in patients with EVT and successful reperfusion [26]. This observation corresponds closely to the findings of lower CBV in the deep MCA territory as it is supplied by lenticulostriate arteries from the M1 segment of the MCA. Collaterals may influence the risk of ICH after EVT. Patients with better collaterals had a lower risk of ICH [27], and collaterals can predict the final infarct volume and clinical outcomes after EVT [27, 28]. Since the M1 segment of the MCA was the most frequent localization of occlusion in our study and ICH occurred most commonly in the deep MCA territory, we assume that the poor collateral status may predispose the deep MCA as a weak point for ICH. A recently published study that evaluated quantitative assessment of maximum CBF of collateral vessels within the Sylvian fissure described that the maximum CBF of collateral vessels was an independent predictor of occurrence of ICH [29]. CTP is only a snapshot of cerebral perfusion as a static evaluation of a multifaceted time-dependent process [15]. Decreases in peri-interventional mean arterial pressure might reduce cerebral blood flow and is associated with worse functional outcome among patients treated under general anaesthesia [30], and hypotension influences outcome in patients not exposed to general anaesthesia [31]. Besides, hypotension before reperfusion may compromise collateral blood flow [31].

This study further confirmed that lower ASPECTS were associated with ICH [12], poor neurological outcomes, and increased mortality after EVT of AIS. The ASPECT Score showed the best effect size for the bleeding prediction in our study, followed by the infarct volume, but overall, the effect sizes vary from no to small effect (Table 3). Both the ASPECTS and the infarct core are easier, faster, and more reliable in assessing daily clinical stroke workflow. Patients with lower ASPECTS and higher infarct core should receive more intensive neurological monitoring. In addition, we found no statistical association between pretreatment with IV-rtPA or carotid stenting and ICH, a finding reported in prior studies [32].

### 5.1. Strengths and Limitations

Some limitations of the present study must be addressed when interpreting the results.

Since our study is a single-center study, it is biased by the choice of the acquisition and analysis of protocol used in our center. The conclusions drawn from our study cannot be generalized for other patients collectively or data processed with other software packages.

Ideally, dual-energy CT would be used to assess the development of ICH. Using monochromatic NCCT, contrast staining can mimic or conceal sICH [33], potentially leading to overestimation of diagnosis of ICH. This point should have been considered in our study due to repeated examinations up to eleven days.

Moreover, a scan duration of >60 s is often recommended to ensure stable results and avoid overestimation of permeability-surface area product values [34]. Nevertheless, using a deconvolution method enables a shorter acquisition time. A 40-second acquisition time was found to be sufficient to preserve an accurate measurement of the permeability-surface area product [35]. First-pass contrast-enhanced PCT was found suitable for assessing microvascular permeability [16].

Furthermore, we did not take time to reperfusion, peri-interventional blood pressure, and collateral status into account in our analysis. Additionally, our cohort is limited by the small sample with <TICI 2B recanalization. Unsuccessful recanalization, defined as TICI grade 2a and below, could contribute to the occurrence of HT [36]. With only 60 patients without successful recanalization, of whom only 8 had PH, this subgroup is relatively small in our study, which could have influenced our statistical results.

As mentioned in the methods, the effect sizes in Table 3 were determined using Cohen's *d*. Since our data is not normally distributed, these results are only to be understood as indicative. Nonetheless, they support the assessment of the practical relevance of the statistically significant results and confirm the assumption that the predictive value of the examined parameters should be assessed as rather low given the large scattering of the results.

Our study is limited by its retrospective design subjecting it to the inherent bias of this analysis type. Bias was mitigated by collecting data prospectively and standardizing thrombectomy techniques and data acquisition.

However, our study has several strengths. It had a relatively large sample size and a larger time interval of follow-up imaging to detect ICH.

This large study cannot fully confirm the results and in particularly the statistic interpretation of previously published results. Therefore, it is absolute necessary even so the studies are published, which did not confirm the expected findings to avoid a bias. ASPECTS and size of initial CTP infarct core were more reliable and useful to assess the risk of ICH after EVT than CT perfusion parameters.

CT perfusion parameters were used in a small effect size related to the occurrence of ICH in AIS but do not reliably improve the prediction of ICH in a clinical useful workflow for decision making in patients considered for acute stroke treatment. While these results on a large cohort are in contrast to other publications, we think it is more than necessary to publish even these results that relay not the promising and hopeful results of other studies and to look critically on the statistics to avoid the “winner's curse.”

## Figures and Tables

**Table 1:** Patient characteristics.

	All patients	Patients without HT	Patients with HI	Patients with PH
Number	392	277	74	41
Age (IQR)	73 (64-80)	73 (64-81)	73 (66-79)	70 (56-79)
NIHSS on admission (IQR)	14 (10-18)	14 (9-18)	15 (10-20)	15 (11-20)
IV-rtPA (%)	168 (42.9)	119 (43)	33 (44.6)	16 (39)
Acute carotid stenting (%)	87 (22.2)	58 (20.9)	16 (21.6)	13 (31.7)
Symptom onset-admission (IQR)	75 (53-177) *N* = 229	74 (50-168) *N* = 172	75 (57-188) *N* = 37	126 (60-226) *N* = 20
Symptom onset-admission (min)	78 (0-650) *N* = 229	112 (0-650) *N* = 172	59 (0-205) *N* = 37	64 (8-215) *N* = 20
Mean systolic pressure	106 (67-167) *N* = 229	105 (73-144) *N* = 172	106 (70-148) *N* = 37	106 (67-167) *N* = 20

Abbreviations: NIHSS, National Institutes of Health Stroke Scale; IV-rtPA, intravenous thrombolysis.

**Table 2:** Radiological data and outcome.

	All patients	Patients without HT	Patients with HI	Patients with PH
Number of patients	392	277	74	41
ASPECTS (IQR)	8 (7-10)	9 (7-10)	8 (6-9)	7 (6-9.5)
Hypoperfusion volume (mL)	118 (67-172)	113 (65-168)	125 (69-184)	130 (78-189)
Ischemic core volume (mL)	5 (0-18)	5 (0-17)	6 (0-18)	7 (4-53)
Successful recanalization (TICI 2b/3) (%)	332 (84.9)	238 (86.2)	61 (82.4)	33 (80.5)
mRS90d (IQR)	3 (2-6)	3 (1-4)	4 (2-6)	5 (4-6)

Abbreviations: ASPECTS, Alberta Stroke Program Early CT Score; TICI, thrombolysis in cerebral infarction; and mRS90d, modified Rankin Scale after 90 days.

**Table 3:** Effect size for bleeding prediction.

	Effect size	Effect size	Effect size
	No HT/HI	No HT/PH	HI/PH
ASPECTS	0.45	0.50	0.07
CTP core	0.09	0.45	0.37
CBF ROI1	0.06	0.21	0.22
CBV ROI1	0.07	0.14	0.23
FED ROI1	0.09	0.16	0.27
CBV 1/1^∗^ ratio^∗^	0.36	0.45	0.14
CBV 1-1^∗^ Diff.^†^	0.11	0.11	0.04
FED 1-1^∗^ Diff.^‡^	0.12	0.11	0.06

Abbreviations: ASPECTS, Alberta Stroke Program Early CT Score; CTP core, computed tomography perfusion infarct core; CBF, cerebral blood flow; CBV, cerebral blood volume; FED, flow extraction product; ROI1, deep middle cerebral artery region of interest. ^∗^Mean values of the CBV in the deep middle cerebral artery ROI of the affected hemisphere relative to the contralateral hemisphere in percent. ^†^Absolute differences between the mean values of the CBV in the deep middle cerebral artery ROI of the affected hemisphere and the contralateral hemisphere. ^‡^Absolute differences between the mean values of the FED in the deep middle cerebral artery ROI of the affected hemisphere and the contralateral hemisphere.

## Data Availability

The data that support the results of this study are available from the corresponding author, upon reasonable request.

## Supplementary Materials

Supplementary Materials Figure I: (A) Time between admission and initial manifestation of HT in the follow-up imaging in days. (B) Time between admission and the largest manifestation of HT in the follow-up imaging in days. HI1: hemorrhagic infarction 1, HI 2: hemorrhagic infarction 2, PH: parenchymal hematoma 1, and PH2: parenchymal hematoma 2. Table I: CTP parameters in the deep middle cerebral artery region of interest. Abbreviations: CBF, cerebral blood flow; CBV, cerebral blood volume; Tmax, maximum enhancement time; FED, flow extraction product; and ROI, region of interest.

## References

[1] Goyal M., Menon B. K., van Zwam W. H. (2016). Endovascular thrombectomy after large-vessel ischaemic stroke: a meta-analysis of individual patient data from five randomised trials. *Lancet*.

[2] Nogueira R. G., Gupta R., Jovin T. G. (2015). Predictors and clinical relevance of hemorrhagic transformation after endovascular therapy for anterior circulation large vessel occlusion strokes: a multicenter retrospective analysis of 1122 patients. *Journal of neurointerventional surgery.*.

[3] Yaghi S., Willey J. Z., Cucchiara B. (2017). Treatment and outcome of hemorrhagic transformation after intravenous alteplase in acute ischemic stroke: a scientific statement for healthcare professionals from the American Heart Association/American Stroke Association. *Stroke*.

[4] Hacke W., Kaste M., Fieschi C. (1998). Randomised double-blind placebo-controlled trial of thrombolytic therapy with intravenous alteplase in acute ischaemic stroke (ECASS II). *The Lancet*.

[5] Balami J. S., White P. M., McMeekin P. J., Ford G. A., Buchan A. M. (2018). Complications of endovascular treatment for acute ischemic stroke: prevention and management. *International Journal of Stroke*.

[6] Albers G. W., Marks M. P., Kemp S. (2018). Thrombectomy for stroke at 6 to 16 hours with selection by perfusion imaging. *New England Journal of Medicine.*.

[7] Nogueira R. G., Jadhav A. P., Haussen D. C. (2018). Thrombectomy 6 to 24 hours after stroke with a mismatch between deficit and infarct. *New England Journal of Medicine.*.

[8] Enomoto Y., Yoshimura S., Egashira Y., Yamagami H., Sakai N., Committee of Endovascular Salvage for Cerebral Ultra-acute Embolism (RESCUE)-Japan Study Group (2016). The risk of intracranial hemorrhage in Japanese patients with acute large vessel occlusion; subanalysis of the RESCUE-Japan registry. *Journal of Stroke and Cerebrovascular Diseases*.

[9] Hao Y., Yang D., Wang H. (2017). Predictors for symptomatic intracranial hemorrhage after endovascular treatment of acute ischemic stroke. *Stroke*.

[10] Paciaroni M., Agnelli G., Corea F. (2008). Early hemorrhagic transformation of brain infarction: rate, predictive factors, and influence on clinical outcome: results of a prospective multicenter study. *Stroke*.

[11] Nael K., Sakai Y., Khatri P., Prestigiacomo C. J., Puig J., Vagal A. (2019). Imaging-based selection for endovascular treatment in stroke. *Radiographics*.

[12] Barber P. A., Demchuk A. M., Zhang J., Buchan A. M. (2000). Validity and reliability of a quantitative computed tomography score in predicting outcome of hyperacute stroke before thrombolytic therapy. *The Lancet*.

[13] Jain A. R., Jain M., Kanthala A. R. (2013). Association of CT perfusion parameters with hemorrhagic transformation in acute ischemic stroke. *AJNR. American Journal of Neuroradiology*.

[14] Aviv R. I., d'Esterre C. D., Murphy B. D. (2009). Hemorrhagic transformation of ischemic stroke: prediction with CT perfusion. *Radiology*.

[15] Renú A., Laredo C., Tudela R. (2015). Brain hemorrhage after endovascular reperfusion therapy of ischemic stroke: a threshold-finding whole-brain perfusion CT study. *Journal of Cerebral Blood Flow and Metabolism*.

[16] Lin K., Kazmi K. S., Law M., Babb J., Peccerelli N., Pramanik B. K. (2007). Measuring elevated microvascular permeability and predicting hemorrhagic transformation in acute ischemic stroke using first-pass dynamic perfusion CT imaging. *AJNR. American Journal of Neuroradiology*.

[17] Kudo K., Sasaki M., Yamada K. (2010). Differences in CT perfusion maps generated by different commercial software: quantitative analysis by using identical source data of acute stroke patients. *Radiology*.

[18] Li Q., Gao X., Yao Z. (2017). Permeability surface of deep middle cerebral artery territory on computed tomographic perfusion predicts hemorrhagic transformation after stroke. *Stroke*.

[19] Patlak C. S., Blasberg R. G., Fenstermacher J. D. (1983). Graphical evaluation of blood-to-brain transfer constants from multiple-time uptake data. *Journal of Cerebral Blood Flow & Metabolism*.

[20] Hacke W., Kaste M., Fieschi C. (1995). Intravenous thrombolysis with recombinant tissue plasminogen activator for acute hemispheric stroke: the European cooperative acute stroke study (ECASS). *Journal of the American Medical Association*.

[21] Leigh R., Jen S. S., Hillis A. E. (2014). Pretreatment blood–brain barrier damage and post-treatment intracranial hemorrhage in patients receiving intravenous tissue-type plasminogen activator. *Stroke*.

[22] Mistry E. A., Mistry A. M., Nakawah M. O. (2017). Mechanical thrombectomy outcomes with and without intravenous thrombolysis in stroke patients: a meta-analysis. *Stroke*.

[23] Kidwell C. S., Latour L., Saver J. L. (2008). Thrombolytic toxicity: blood brain barrier disruption in human ischemic stroke. *Cerebrovascular diseases.*.

[24] Batchelor C., Pordeli P., d’Esterre C. D. (2017). Use of noncontrast computed tomography and computed tomographic perfusion in predicting intracerebral hemorrhage after intravenous alteplase therapy. *Stroke*.

[25] Suh C. H., Jung S. C., Cho S. J. (2019). Perfusion CT for prediction of hemorrhagic transformation in acute ischemic stroke: a systematic review and meta-analysis. *European radiology.*.

[26] Arenillas J. F., Cortijo E., García-Bermejo P. (2018). Relative cerebral blood volume is associated with collateral status and infarct growth in stroke patients in SWIFT PRIME. *Journal of Cerebral Blood Flow & Metabolism.*.

[27] Bang O. Y., Saver J. L., Kim S. J. (2011). Collateral flow averts hemorrhagic transformation after endovascular therapy for acute ischemic stroke. *Stroke*.

[28] Shuaib A., Butcher K., Mohammad A. A., Saqqur M., Liebeskind D. S. (2011). Collateral blood vessels in acute ischaemic stroke: a potential therapeutic target. *The Lancet Neurology.*.

[29] Shi F., Gong X., Liu C. (2019). Acute stroke: prognostic value of quantitative collateral assessment at perfusion CT. *Radiology*.

[30] Treurniet K. M., Berkhemer O. A., Immink R. V. (2018). A decrease in blood pressure is associated with unfavorable outcome in patients undergoing thrombectomy under general anesthesia. *Journal of Neurointerventional Surgery*.

[31] Whalin M., Halenda K., Haussen D. (2017). Even small decreases in blood pressure during conscious sedation affect clinical outcome after stroke thrombectomy: an analysis of hemodynamic thresholds. *American Journal of Neuroradiology*.

[32] Katsanos A. H., Tsivgoulis G. (2019). Is intravenous thrombolysis still necessary in patients who undergo mechanical thrombectomy?. *Current Opinion in Neurology*.

[33] Almqvist H., Holmin S., Mazya M. V. (2019). Dual energy CT after stroke thrombectomy alters assessment of hemorrhagic complications. *Neurology*.

[34] Yeung T. P. C., Yartsev S., Bauman G., He W., Fainardi E., Lee T.-Y. (2013). The effect of scan duration on the measurement of perfusion parameters in CT perfusion studies of brain tumors. *Academic radiology.*.

[35] Mazzei F. G., Volterrani L., Guerrini S. (2014). Reduced time CT perfusion acquisitions are sufficient to measure the permeability surface area product with a deconvolution method. *BioMed Research International*.

[36] Wang D. T., Churilov L., Dowling R., Mitchell P., Yan B. (2015). Successful recanalization post endovascular therapy is associated with a decreased risk of intracranial haemorrhage: a retrospective study. *BMC Neurology*.

