# Supplementary material for: Value of Perfusion CT in the Prediction of Intracerebral Hemorrhage after Endovascular Treatment
Source: Stroke Res Treat. 2021 Jul 22;2021:9933015. doi: 10.1155/2021/9933015 (PMC8321751; doi:10.1155/2021/9933015)
Supplement: Supplementary Materials — Figure I: (A) Time between admission and initial manifestation of HT in the follow-up imaging in days. (B) Time between admission and the largest manifestation of HT in the follow-up imaging in days. HI1: hemorrhagic infarction 1, HI 2: hemorrhagic infarction 2, PH: parenchymal hematoma 1, and PH2: parenchymal hematoma 2. Table I: CTP parameters in the deep middle cerebral artery region of interest. Abbreviations: CBF, cerebral blood flow; CBV, cerebral blood volume; Tmax, maximum enhancement time; FED, flow extraction product; and ROI, region of interest. [file 9933015.f1.docx]

***Online-supplement***

**Value of Perfusion CT in Prediction of Intracerebral Hemorrhage after Endovascular Treatment**

Friederike Austein, MD ^[[1]](#footnote-1)^^[[2]](#footnote-2)^*, Antonia Carlotta Fischer^[[3]](#footnote-3)^, Jens Fiehler, Prof.^1^,

Olav Jansen, Prof.^2^, Thomas Lindner, PhD^1^, Susanne Gellißen, MD^1^

**S1**

**Package A Syngo CT:**

To account for the different constitution of brain tissue depending on the anatomic localization, we measured CTP parameters in regions of interest (ROIs) referring to the ASPECTS regions.

For each patient, freehand ROIs were defined according to the ASPECTS regions on the stroke-affected hemisphere. In contrast to the ASPECTS regions, the deep middle cerebral artery (MCA) territory was summarized in one ROI after previous authors made the observation that patients with proximal LVO occasionally develop ICH in the affected deep MCA territory after recanalization therapy.

The software automatically calculated mirrored ROIs on the contralateral hemisphere. For each ROI, both on the affected and the contralateral hemisphere, quantitative values were measured for CBF, CBV, mean transit time (MTT), maximum enhancement time (Tmax) and FED. Additionally, the ratios and absolute differences between the ROIs were calculated. The CTP values of the affected hemisphere could thereby be evaluated relative to the normal brain tissue on the contralateral hemisphere to consider potential interpatient variability. In this study, we assessed CBF, CBV, Tmax and FED.

The Patlak(1) model, which was based on a two-compartment model was used for assessing the FED. Patlak analysis uses a two-compartment model that describes the one-way transfer of contrast material from the intravascular space to the extravascular space. At any timepoint, the tissue concentration of contrast material is equivalent to the sum of the intravascular and extravascular concentrations of contrast material as denoted by the following equation:

C(t) = BV ∙ b(t) + K ∫ b(t)dt,

where C(t) is the concentration of contrast material within the tissue, BV is the blood volume, b(t) is the concentration of contrast material in blood, and K is the volume transfer constant. Dividing the equation by b(t) produces the linear relationship

C(t)/b(t) = BV + K ∫ b(t)dt/b(t).

By fitting a straight line to the data points, K can be derived from the slope of this line and BV from the intercept.

K describes the portion of blood flow F that is extracted into the extravascular space, K = E ∙ F, with the extraction fraction E, which is defined by the following equation:

E = (1 − exp[PS/F(1 − Hct)] ,

where PS is the permeability–surface area product and Hct the hematocrit value.

**S2**

**Package B RAPID:**

Volumes of ischemic core and hypoperfused tissue were estimated fully automatically. RAPID defines the ischemic core as tissue not exceeding a threshold of <30% in CBF relative to normal tissue and hypoperfused regions (Tmax > 6s).


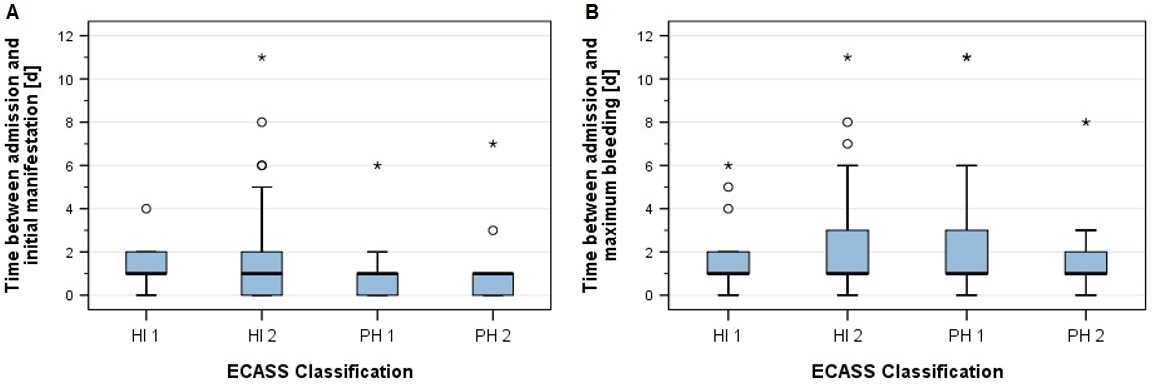


**Supplement Figure I.**

**A.** Time between admission and initial manifestation of HT in the follow-up-imaging in days **B.** Time between admission and the largest manifestation of HT in the follow-up imaging in days.

HI1 = Hemorrhagic Infarction 1, HI 2 = Hemorrhagic Infarction 2, PH = Parenchymal Hematoma 1, PH2 = Parenchymal Hematoma 2.

|  | Patients without HT | Patients with HI | Patients with PH |
| --- | --- | --- | --- |
| CBF 1 [mL/100mL/min] † | 38.05 (27.82/50.25) | 32.96 (25.83/49.97) | 32.35 (24.3/45.91) |
| CBF 1* [mL/100mL/min] ‡ | 62.98 (56.94/68.57) | 63.92 (57.3/69.39) | 64.30 (58.23/67.2) |
| CBF 1/1* ratio [%] § | 62.92 (46.44/78.66) | 55.54 (41.13/73.61) | 49.99 (41.59/69.18) |
| CBF 1-1* Diff. [mL/100mL/min] \|\| | -23.01(-32.63/-13.34) | -27.39 (-35.25/-16.82) | -30.83 (-38.31/-20.38) |
| CBV 1 [mL/100mL] † | 2.82 (2.34/3.39) | 2.57 (1.97/3.16) | 2.57 (1.9-3.14) |
| CBV 1* [mL/100mL] ‡ | 3.35 (3.14/3.57) | 3.36 (3.15/3.61) | 3.34 (3.19/3.57) |
| CBV 1/1* ratio [%] § | 85.82 (71.26/98.79) | 76.10 (62.02/93.18) | 76.77 (56.56/86.59) |
| CBV 1-1* Diff. [mL/100mL/min]  \|\| | -0.49 (-0.96/-0.05) | -0.80 (-1.34/-0.24) | -0.81 (-1.77/-0.48) |
| TMAX 1 [s] † | 4.05 (2.4/6.03) | 4.94 (3.13/6.33) | 4.59 (3.32/6.58) |
| TMAX 1* [s] ‡ | 0.33 (0.05/1) | 0.57 (0.08/1.13) | 0.40 (0.04/1.15) |
| TMAX 1/1* ratio [%] § | 910.00 (425/4425) | 718.03 (363.37/3862.5) | 1307.02 (390.77/5700) |
| TMAX 1-1* Diff. [s] \|\| | 3.41 (1.9/5.32) | 4.04 (2.42/5.36) | 3.78 (3.1/5.25) |
| FED 1 [mL/100mL/min] † | 1.83 (1.31/2.48) | 1.61 (1.17/2.26) | 1.64 (1.1/2.56) |
| FED 1* [mL/100mL/min] ‡ | 0.68 (0.45/0.95) | 0.73 (0.46/1.14) | 0.66 (0.38/1.04) |
| FED 1/1* ratio [%] § | 265.93 (172.48/431.09) | 226.46 (142.33/348.57) | 224.53 (146.7/355.66) |
| FED 1-1* Diff. [mL/100mL/min] \|\| | 1.07 (0.57/1.84) | 0.90 (0.44/1.19) | 0.77 (0.38/1.29) |

**Supplement Table I.** CTP Parameters in the deep middle cerebral artery region of interest

Abbreviations: CBF, cerebral blood flow; CBV, cerebral blood volume; Tmax, maximum enhancement time; FED, flow extraction product; ROI, region of interest

† Mean values in the deep middle cerebral artery ROI of the affected hemisphere

‡ Mean values in the deep middle cerebral artery ROI of the contralateral hemisphere

§ Mean values in the deep middle cerebral artery ROI of the affected hemisphere relative to the contralateral hemisphere in percent

|| Absolute differences between the mean values in the deep middle cerebral artery ROI of the affected hemisphere and the contralateral hemisphere

References

1. Patlak CS, Blasberg RG, Fenstermacher JD. Graphical evaluation of blood-to-brain transfer constants from multiple-time uptake data. Journal of Cerebral Blood Flow & Metabolism. 1983;3(1):1-7.

1. Department for Diagnostic and Interventional Neuroradiology, University Medical Center Hamburg-Eppendorf, Hamburg, Germany [↑](#footnote-ref-1)
2. * Corresponding author. E-mail: f.austein @uke.de; Phone: +4915222816068; Fax: +4940741040114 [↑](#footnote-ref-2)
3. Department of Radiology and Neuroradiology, University Hospital Schleswig-Holstein, Germany [↑](#footnote-ref-3)
